# Integrating Sensors into a Marine Drone for Bathymetric 3D Surveys in Shallow Waters

**DOI:** 10.3390/s16010041

**Published:** 2015-12-29

**Authors:** Francesco Giordano, Gaia Mattei, Claudio Parente, Francesco Peluso, Raffaele Santamaria

**Affiliations:** Dipartimento di Scienze e Tecnologie, Università degli Studi di Napoli "Parthenope", Centro Direzionale, Isola C4, 80143 Napoli, Italy; francesco.giordano@uniparthenope.it (F.G.); claudio.parente@uniparthenope.it (C.P.); francesco.peluso@uniparthenope.it (F.P.); raffaele.santamaria@uniparthenope.it (R.S.)

**Keywords:** marine USV, open prototype, bathymetry, shallow waters, Gegraphic Iinformation System (GIS) application, 3D bathymetric data elaboration

## Abstract

This paper demonstrates that accurate data concerning bathymetry as well as environmental conditions in shallow waters can be acquired using sensors that are integrated into the same marine vehicle. An open prototype of an unmanned surface vessel (USV) named MicroVeGA is described. The focus is on the main instruments installed on-board: a differential Global Position System (GPS) system and single beam echo sounder; inertial platform for attitude control; ultrasound obstacle-detection system with temperature control system; emerged and submerged video acquisition system. The results of two cases study are presented, both concerning areas (Sorrento Marina Grande and Marechiaro Harbour, both in the Gulf of Naples) characterized by a coastal physiography that impedes the execution of a bathymetric survey with traditional boats. In addition, those areas are critical because of the presence of submerged archaeological remains that produce rapid changes in depth values. The experiments confirm that the integration of the sensors improves the instruments’ performance and survey accuracy.

## 1. Introduction

Bathymetric information is fundamental in all branches of oceanography, paleoclimate studies, and marine geology. It can be supplied by maps that indicate the water body depth as a function of the position (latitude and longitude), similar to topographic maps representing the altitude of the Earth’s surface at different geographic coordinates [1].

Most techniques for obtaining these data are difficult to use in shallow waters where bathymetric surveys often entail expensive measurement costs . For most bathymetry acquisition techniques, it is not possible to obtain a better vertical accuracy than 0.5 m at the 95% confidence level. Airborne LiDAR and/or maritime vessels are the only options for surveys with an accuracy requirement of 0.5 m with a 95% confidence level. Other remote sensing techniques can also be used only if the accuracy requirements are relaxed to 2 m, 95% confidence [2].

Airborne laser (or lidar) bathymetry (ALB) is based on a scanning, pulsed laser beam to measure the depths of relatively shallow, coastal waters from the air. It is also named airborne lidar hydrography (ALH) when used principally for nautical charting [3].

The use of maritime vessels capable of carrying out bathymetric measurements is limited by the depth of the waters, so only small crafts are suitable in shallow waters. Because of their reduced dimensions, these vessels are not manned and are categorized as USVs (Unmanned Surface Vehicles) [4,5]. By analogy with avionics applications, they are also called marine drones [6]. Some such drones are also known as Autonomous Surface Crafts (ASCs) and Remotely Operated Vehicles (ROVs). According to [7], ASCs, also called autonomous surface vehicle (ASVs), are a kind of autonomous marine vehicle without the direct operation of humans, while ROVs are controlled by an operator who is not on-board. However, this distinction is not always observed and the terms are sometimes used with no difference in meaning.

In the last few years several specific crafts have been built for surveying in shallow waters, as reported in the literature.

In June 2006, the US Geological Survey Woods Hole Science Center (WHSC) integrated an ASV for hydrographic surveys in shallow waters (1–5 m), which was designed to map seafloor morphology and surficial sediment distribution and thickness. Named the Independently (or) Remotely Influenced Surveyor (IRIS) and designed as a catamaran-based platform (10 feet in length, 4 feet in width, and approximately 260 lbs in weight), this vehicle is equipped with a chirp dual-frequency side scan sonar (100/500 kHz) and seismic-reflection profiler (4–24 kHz), a wireless video camera and single-beam echosounder (235 kHz). IRIS is operated remotely through a wireless modem network enabling the real-time monitoring of data acquisition and navigated using RTK [8].

The ROAZ unmanned surface vehicle was proposed by the Autonomous Systems Laboratory (ASL) from Porto Polytechnic Institute (ISEP) for marine operations. It was designed to work in very shallow rivers and marine coastlines. Because of the possibility of transmitting the entire data collection on-board a base station, the operator receives online feedback on the vehicle’s location and performance, as well as side-scan sonar imagery and bathymetry quality [9].

Another example of a craft used for bathymetric surveys in shallow water was developed by the Underwater Robotic Research Group’s (URRG) who developed the URG—ASV, a battery-powered vessel [10].

CatOne is an example of a catamaran-robot that can operate in very shallow waters as well as in sensitive ecosystems because of its very low draft and an electric propulsion that guarantees zero pollution emission and low noise. It carries sonar and GPS on-board and can be equipped with other sensors to support different activities such as environment monitoring [11].

The purpose of this research was to create a marine drone, optimized for surveys in very shallow water, and benefitting from previous experiences in this field as noted above. The innovation of this project is twofold. First, the data and video are broadcast directly to several operators, enabling the visualization and the pre-processing of all data in real time, by means of several devices managed by experts from different disciplines (such as an archaeologist, a geophysicist, a topographer or a GIS expert). This feature was implemented in order to carry out interdisciplinary surveys in critical coastal areas In fact, in the two study cases (both in the Gulf of Naples) there are submerged archaeological remains in the survey area. Thus, each expert can verify that the data acquisition is correct from his/her point of view. In addition, in order to also obtain high precision bathymetric data in critical areas, a system of data quality control was implemented, using an inertial platform.

## 2. Experimental Section

The MicroVeGA drone is an Open Project of USV conceived, designed and built to operate in shallow water areas (0–20 m), where a traditional boat is poorly manoeuvrable. It was engineered by the DIST research group at the University of Naples and was designed to test the procedures and methods of morpho-bathymetric surveys in critical areas. In [7], the initial development phase of MicroVeGA is described.

The drone is a small and ultra-light catamaran that can be assembled in 30 min, with a few draught centimetres, therefore suitable to perform surveys up to the shoreline. It is driven by non-polluting electric motors, and is therefore suitable to perform surveys in marine protected areas. Table 1 lists the characteristics.

**Table 1:** Technical and physical characteristics of the drone.

Characteristics	Measures
Overall length	135 cm
Width	85 cm
Weight in navigation trim	20 kg
Motors	2 brushless 750 kV/140 W
Operating speed	0.5–2 m/s
Power autonomy	2–4 h

**Figure 1:**
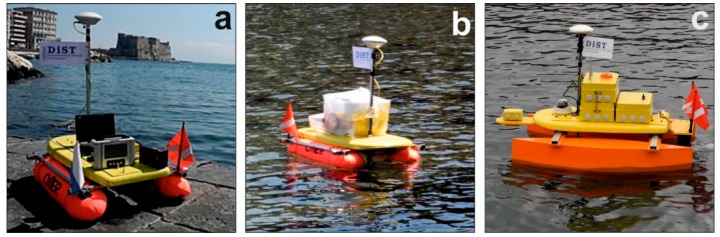
(**a**) Instruments on board of MicroVeGA; (**b**) Prototype #1 of MicroVeGA; (**c**) Prototype #2 of MicroVeGA.

MicroVeGA is an evolving open project that has enabled surveys to be carried out already in its early stage of development (Figure 1). In this paper, two study cases are illustrated, the first created with the MicroVeGA prototype #1 (Figure 1b), the second with the MicroVeGA prototype #2 (Figure 1c).

**Figure 2:**
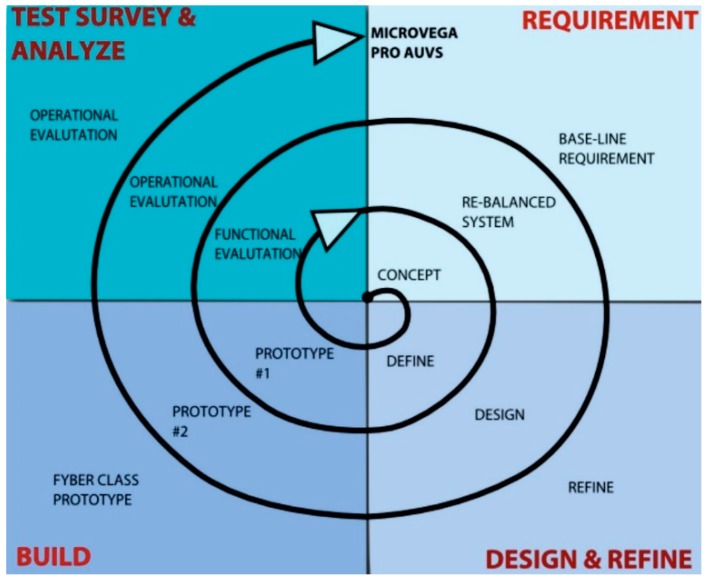
Spiral model of the project management.

This project is a low risk technology project. The spiral model of project management [12] is divided into smaller sections (Figure 2). Each prototype requires the following phases: requirements, design and refine, build; test, survey and analyse.

The current version of MicroVeGA (*i.e.*, Prototype #2) is remotely controlled by an operator and is equipped with a set of sensors for acquiring morpho-bathymetric high-precision data (see Section 2.2).

### 2.1. System Architecture

**Figure 3:**
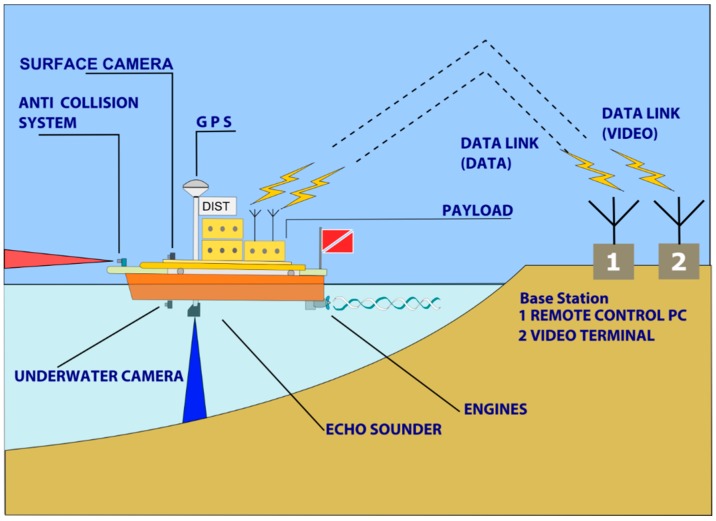
Data acquisition architecture: a base station, with a remote control PC and a video terminal; an on board computerized system that manages the on-board instrumentation; a communication system via data link, to connect the AUVS with the base station.

The architecture of the data acquisition system (Figure 3) includes: (i) a base station, with a remote controlled PC and a video terminal; (ii) an on-board computerized system that manages the on-board instrumentation; (iii) a communication system via data link, to connect the UVS with the base station.

The operator responsible for the base station manages the mission data by means of TrackStar software by defining the navigation routes and monitoring the mission progress. The TrackStar software (described in Section 2.3 Data Acquisition and Software), implemented by our research group, manages the survey activities and automatically creates a measurement geodatabase.

The data is stored on board in RAW format by a computerized system that acquires and organizes the GPS, echo sounder, inertial platform, and obstacle-detection sensor data. This data is broadcast to the base station by a data link system, after which several operators can simultaneously receive the data in real time.

MicroVeGA data transmission is based on two wireless networks. The first transmits the telemetry data (*i.e.*, position, depth, atmospheric temperature and obstacle detection) from the vessel to Trackstar. The second network transmits the videos of the two on-board cameras to the base station. This information is managed by a specific app, and the images are viewed on a tablet in real time.

### 2.2. Sensors and Methods for Data Acquisition

**Figure 4:**
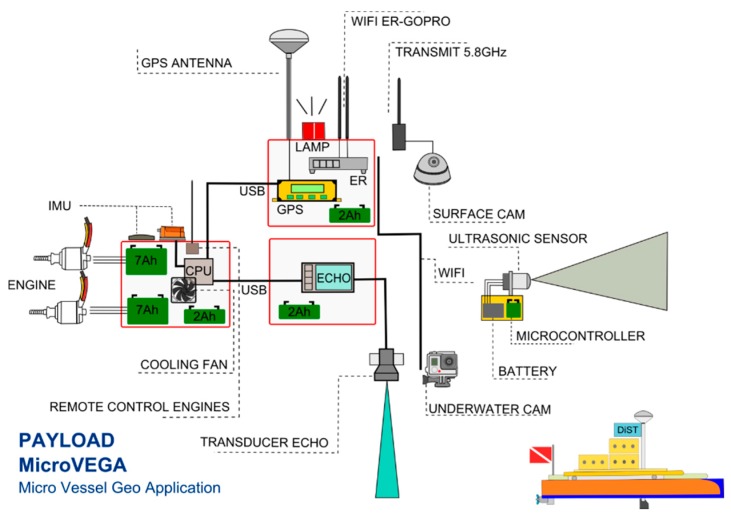
Payload of MicroVeGA drone.

The main instruments on-board are: (1) microcomputer; (2) differential GPS system and Single beam echo sounder; (3) integrated system for attitude control; (4) obstacle-detection system (SIROS1) with temperature control system; (5) video acquisition system (both above and below sea level) (Figure 4).

#### 2.2.1. Microcomputer

An OLinuXino microcomputer, with a Linux operating system, and three high-speed serials, manages all the survey phases, the data recording and its wi-fi transmission to the base station. An Arduino microcontroller controls the drone’s engines, the temperature measurements, and the management of the obstacle-detection ultrasound systems (Figure 4).

#### 2.2.2. GPS and Single Beam Echo Sounder (SBES)

**Figure 5:**
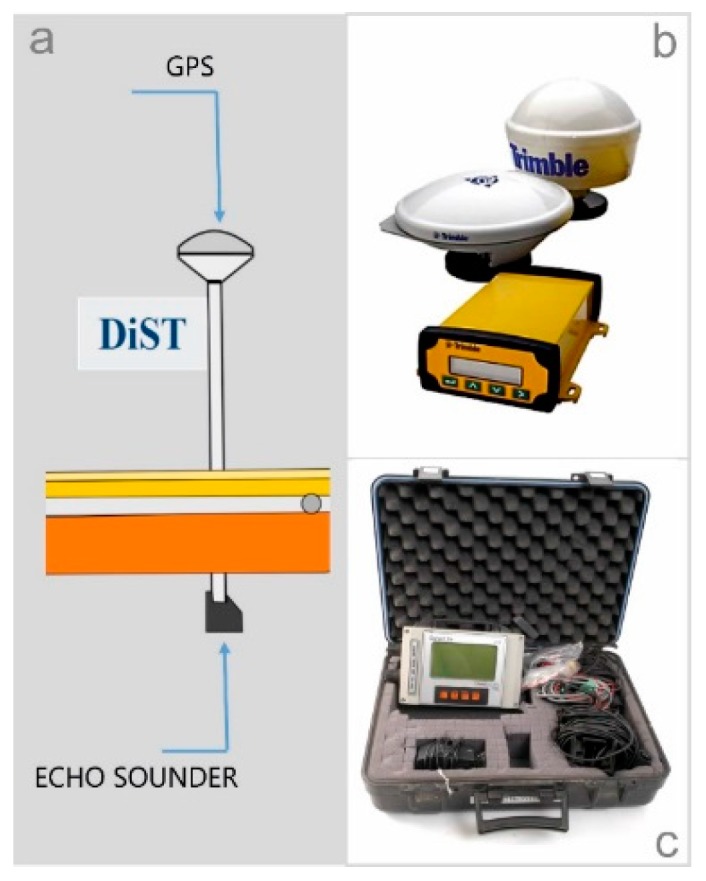
(**a**) Installation positions of the GPS and echo sounder on drone; (**b**) Trimble DSM232 GPS; (**c**) Omex Sonarlite echo sounder.

The GPS receiver (Figure 5b), installed on board MicroVeGA, is the Trimble DSM™ 232 (24-channel L1/L2), which is a robust solution for dynamic positioning tasks in the marine environment. In fact, this device is easily installed and is able to withstand tough environmental conditions, and is thus suitable for surveys in very shallow waters. In addition, the GPS receiver and antenna are modular, and thus it was possible to install on board of MicroVeGA, the antenna vertically with respect to the SBES transducer.

The Trimble DSM 232 GPS receiver enables the appropriate GPS correction method and accuracy to be selected. In this research, the DGPS option in post-processing was used, using Trimble software.

The SonarLite (Omex) is the SBES installed on-board (Figure 5c). This instrument is optimized for the bathymetric survey in shallow waters, and its transducer is positioned vertically above the GPS receiver in order to remove any offset (Figure 5a).

#### 2.2.3. Inertial Platform Unit (IMU)

The inertial measurement unit used for measuring balance and direction on board of MicroVeGA is the Xsense MTi series G. This device is an integrated GPS and MEMS IMU with a Navigation and Attitude and Heading Reference System processor. It was used on the MicroVeGA drone because of it weighs very little.

The internal low-power signal processor runs a real-time Xsens Kalman Filter (XKF), providing inertial enhanced 3D position and velocity estimates [13,14].

**Figure 6:**
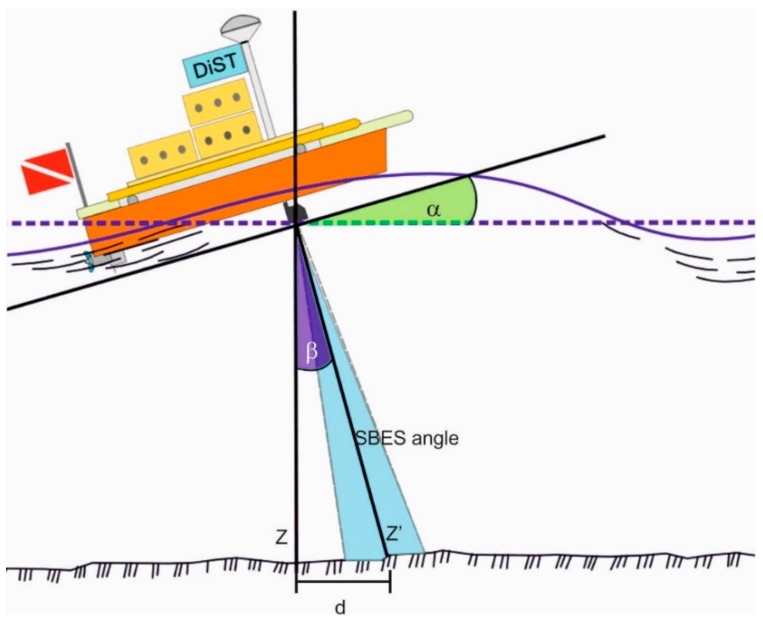
Horizontal error due to pitch or roll.

The IMU data are stored in the survey geodatabase and increase the accuracy of the survey since measurements affected by attitude errors are removed [15]. In the case of errors due to pitch and roll, a quality control system that removes all measurements higher than a specific limit d was implemented (Figure 6):

(1) d≤spp

where:

(2) $$d=Z^{\prime }\;\text{sin}\;\beta$$

and Z′ = echo sounder measurement; *spp* = survey parameter precision related to survey scale, depth, survey target; β = angle between Z and Z′ = 90° − (90° − α); α = pitch or roll.

In the planning phase of the survey, the operator can establish the value of the spp survey parameter, thus defining the horizontal limit d that makes a measurement valid.

**Figure 7:**
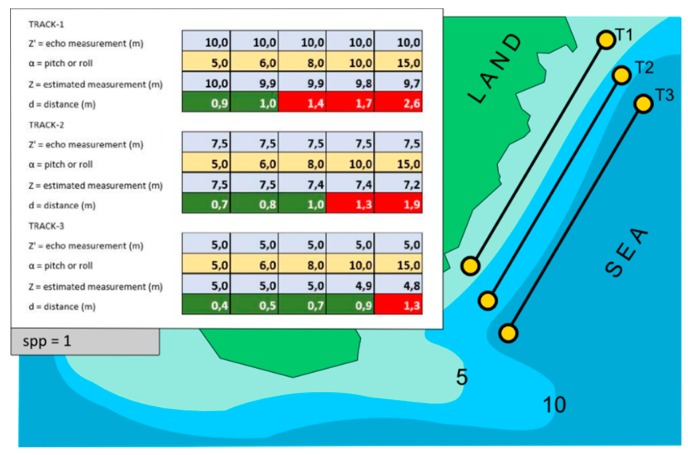
Example of three data acquisitions with spp = 1 constant and with α and Z′ variables.

In both surveys described below (archaeological survey with rocky seabed and a cartographic scale of 1:1000), the threshold value spp was set equal to 1. As shown in Figure 7, if the measured depth increases, the roll angle becomes even more critical. In fact the same angle of roll (or pitch), equal to 10°, is associated with a valid measurement if the depth is −5 m, while it is associated with an invalid measurement if the depth is greater than −7 m (see also Table 2).

As the weather and sea conditions are essential for the proper execution of a bathymetric survey, surveys are not normally carried out when waves are beyond a certain strength. The validation system is primarily to prevent the storage of incorrect data due to occasional events, such as the passage of a vessel, and thus to improve the quality level of the whole survey.

**Table 2:** Variation of the distance d with the changing depth (see also Figure 7).

Measurement Parameters	T1	T2	T3
Z′ = Echo Measurement (m)	5.0	7.5	10.0
α = Pitch (or roll)	10.0	10.0	10.0
Z = Estimated Measurement (m)	4.9	7.4	9.8
d = distance (m)	0.9	1.3	1.7

The mission software—Trackstar—manages these calculations in real time highlighting the invalid measurements with a special color scale. This visualization allows the operator to evaluate the areal coverage of the survey, and to decide the possible repetition of a navigation line in real time.

**Figure 8:**
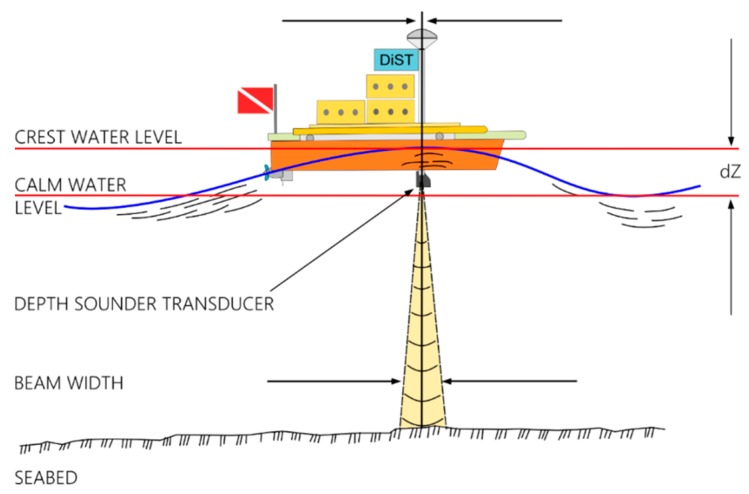
Vertical error in depth measuring due to the wave effect.

The IMU data are also used to correct the depth with respect to the vertical error due to the wave effect (Figure 8):

(3) CWL=Z±dZ

where: *CWL* = clam water level; *Z* = depth measured by SBES; *dZ* = vertical error measured by inertial platform.

#### 2.2.4. SIROS 1 (Obstacle-Detection System—In Italian: Sistema Rilevamento OStacoli)

**Figure 9:**
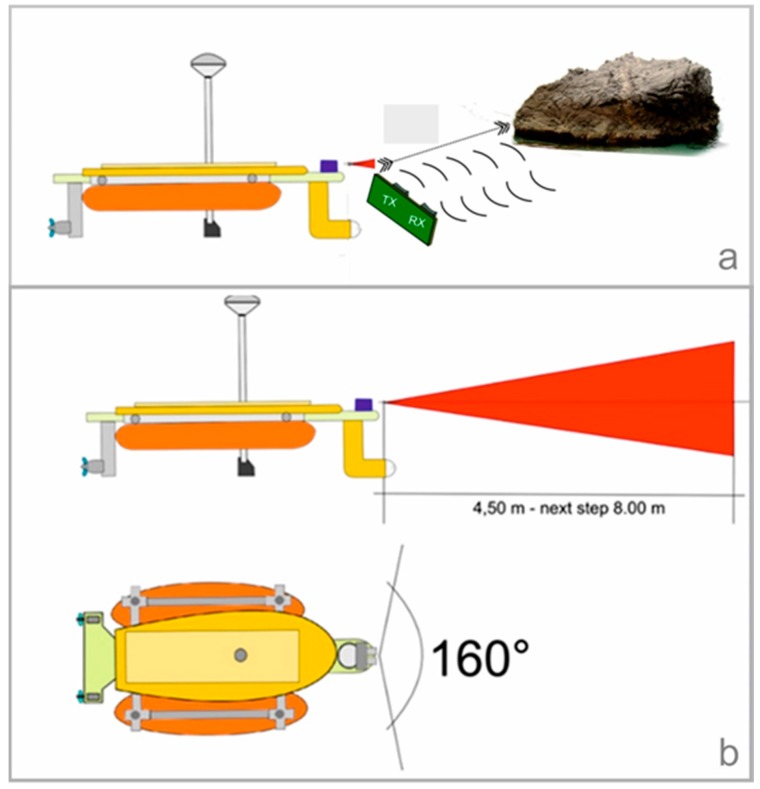
(**a**) Operation of the ultrasonic sensor; (**b**) Action range of obstacle-detection system.

The system is based on: an Arduino controller; an ultrasonic sensor; a temperature sensor; a servomechanism; an electronic component; and a software application. The main sensor used is the HY-SR05, which is able to detect emerged obstacles in the range of 2–450 cm, with an accuracy of 0.2 cm. The HY-SR05 uses a single output pin on the controller to send a trigger pulse to the sensor, and then another input pin to receive the pulse indicating the object’s distance (Figure 9a).

Using a servomechanism, the obstacle-detection system can scan a prow sector of about 160° (Figure 9b). The software controls the ultrasonic sensor and using the servomechanism moves the azimuth of the same sensor in steps of 5°.

The distance detection is a function of the air temperature, and the obstacle-detection system is equipped with a temperature sensor (LM35) that compensates for temperature variations with an accuracy of ±0.5 °C, making the obstacle-detection system more efficient.

According to the Laplace law, in the case of the air the speed of sound increases by 0.6 m/s for each increase of 1 °C air temperature:

(4) $$v=331.3\frac{m}{s}+0.606\;T_{i}$$

where *v* = sound velocity in the air; 331.5 m/s = the sound velocity at 0 °C; *T*_i_ = Air temperature value in a specified measure time.

Table 3 demonstrates the increasing accuracy of the measurements (dD column), by a comparison between the distance measured at the standard temperature of 20 °C (columns V1 and D1), and the distance measured at the current temperature (columns V2 (t) and D2), showing how the variation in the temperature influences the measured values.

**Table 3:** Comparison table between the distance measured at the standard temperature of 20 °C (columns V1 and D1), and the distance measured at the actual temperature (columns V2 (t) and D2).

V1 (20°) m/s	T (°C)	V2 (T) m/s	Time (s)	D1 (cm)	D2 (cm)	dD (cm)
343.4	5	334.3	0.010	171.7	167.2	4.5
343.4	10	337.4	0.010	171.7	168.7	3.0
343.4	20	343.4	0.010	171.7	171.7	0.0
343.4	30	349.5	0.010	171.7	174.7	−3.0

The obstacle-detection system, along with the camera’s surface, is very useful when there are obstacles, such as scattered rocks, that are not marked on the cartography. This system enables the operator to navigate up to a few centimeters from the docks and piers, and thus is very useful in bathymetric surveys carried out in ports and harbours.

SIROS becomes active when the distance from an obstacle is <400 cm. As soon as this happens, TrackStar displays the progressive distances of the obstacle, thus alerting the operator. Normally the operator decreases the speed and, if necessary changes route. An operator controls the MicroVeGA drone remotely, and thus there are no automatic collision avoidance maneuvers. The only automatic actions of the system are:

- activate alarm visual and sound software management and control,
- activate flashing and sirens on board.

Especially in critical cases, the software automatically stops the engines and activates an alarm (go home command) to warn the operator about the need to stop the mission. Finally, SIROS 1 has a safety navigation system to support the operator in making browsing simpler, safer and fast.

#### 2.2.5. Video Acquisition System

MicroVeGA has a complete system for video data acquisition, above and below sea level. Two GO PRO HERO 3 cameras are installed on-board, one above the water level and the other below. The cameras make a video recording during the whole survey, enabling the operator to check the environmental conditions and to manage the presence of obstacles in real-time. Video data is transmitted to the base station and is recorded on a hard disk.

For performance testing, two methods for transmitting video data from on board to the base station were used. One uses the wi-fi on board a GoPRO camera that (thanks to the Extended Range WiFi positioned on the MicroVeGA) transmits data to the shore. Here any wi-fi device (smart phone, tablet, or PC) can view content in realtime thanks to the app supplied with the GoPRO. The second method uses a 5.8 GHz 100 mW 8 channel video transmitter along with a RC805 5.8 GHz AV Receiver. A small LCD, connected to the receiver, displays real-time video. In the next version of the drone, the second solution will be used, *i.e.*, without the Go-Pro wi-fi, as this will ensure low weight, the high flow rate, and the availability of more transmission channels.

### 2.3. Data Acquisition and Software

The TrackStar software, developed by our research group, manages the survey activities and automatically creates a measurement geodatabase.

**Figure 10:**
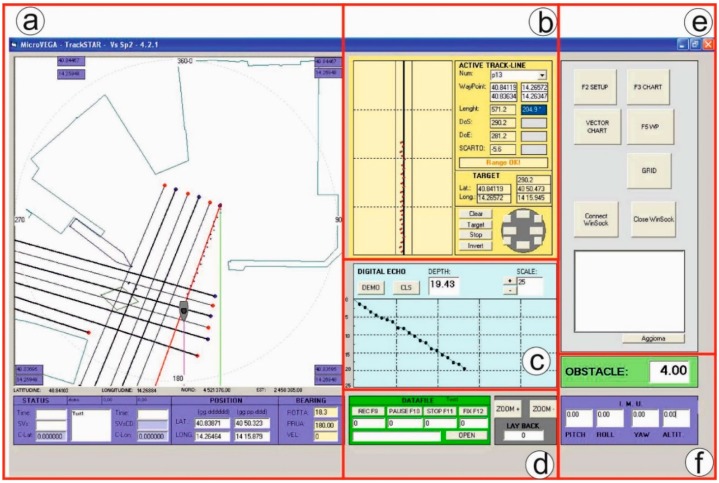
Trackstar desktop: (**a**) real time navigation; (**b**) deviation of the vessel from the planned line; (**c**) real time bathymetric profile; (**d**) real time data recording and datafile creation; (**e**) import of cartography and creation of navigation lines; (**f**) obstacle distance and attitude measurements.

The software displays in real time (Figure 10): the GPS navigation; the deviation of the vessel from the planned line; the SBES bathymetric measurements along the navigation line (bathymetric profiles); the distance from a detected obstacle; and the IMU measurements (pitch, roll, yaw and altitude).

The software also displays the data read from the IMU and, near to an emerged obstacle, shows the distance from the obstacle to the drone, thus facilitating the remote control of operations by the operator. All data from GPS, SBES and IMU are stored in a single datafile in ASCII format. The software was developed in Windows.

## 3. Results and Discussion

This section describes two cases of the MicroVeGA survey. The main characteristic of these areas is the coastal physiography that prevents any bathymetric surveys with traditional boats. There are also submerged archaeological remains that produce rapid changes in depth values.

The morpho-bathymetric survey carried out in each area was planned in order to obtain a GIS 3D model of the sea floor. The interpolation method used in the post-processing phase was the Inverse Distance Weighted (IDW) interpolation. This interpolator is one of the simplest and most readily available methods for interpolation. It is based on an assumption that the value at an unsampled point can be approximated as a weighted average of values at points within a certain cut-off distance, or from a given number of the closest points [16].

### 3.1. MicroVeGA Survey 1

The first bathymetric survey of MicroVeGA drone was carried out along the Sorrento Marina Grande coast in the nearshore area (0–3 m depths) between the tufa cliff and coastal protection works using Prototype #1. In Prototype #1 of the drone, the instruments were all contained in a plexiglas non waterproof case and the hulls of the catamaran consisted of two float tubes.

**Figure 11:**
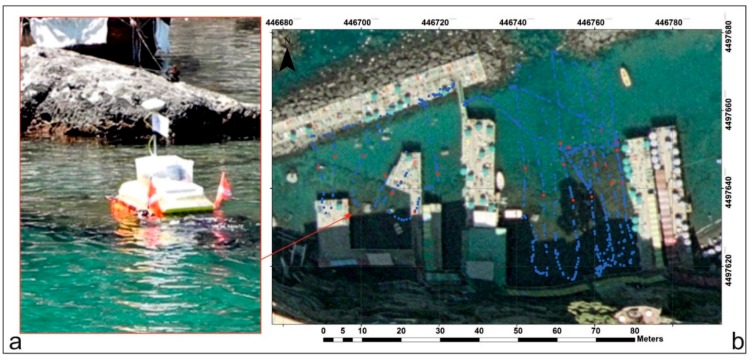
(**a**) MicroVeGA drone prototype #1 used during the survey; (**b**) navigation lines of bathymetric survey in blue and position of archaeological targets located by submerged camera and SBES in red.

The navigation of the bathymetric survey (Figure 11) had a linear development of about 500 m, with a distance between the navigation lines of about 2 m. In the first instance, the positioning and the morphologic reconstruction were obtained of all the archaeological remains in the area [17], using the GPS, SBES and submerged camera.

**Figure 12:**
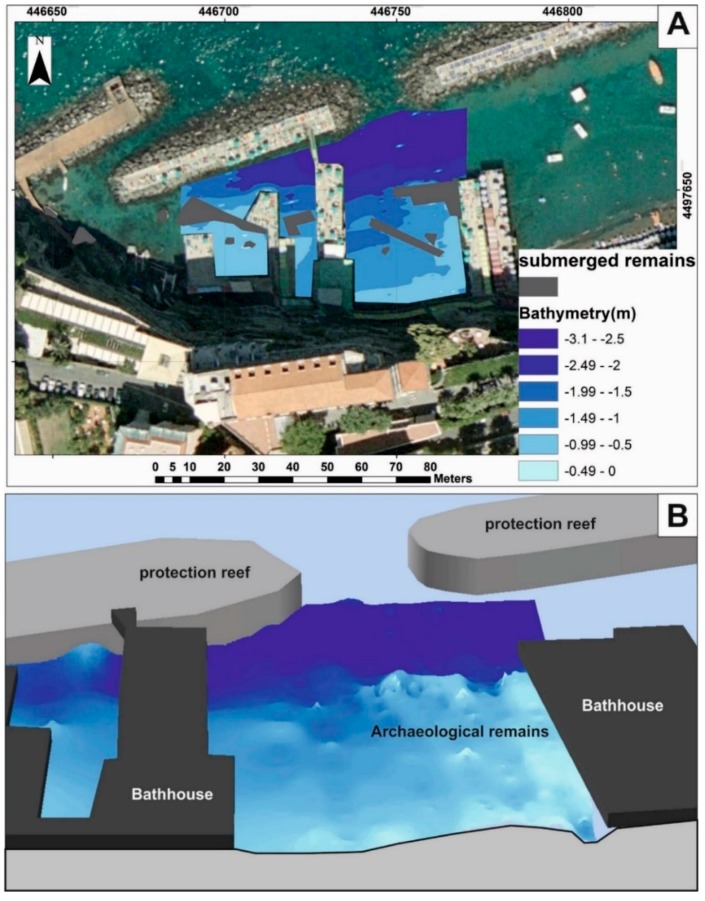
(**a**) 2D visualization of the sea floor digital model of the study area—Sorrento Marina Grande (Naples, Italy); (**b**) 3D visualization of the same sea floor.

In addition, 3D data were processed in the ARCGIS environment, using 3D Analyst. The interpolation of the bathymetric data, through the IDW interpolator, transformed the point measurements into continuous measurements. The final product is a seafloor digital model of the area (Figure 12).

### 3.2. MicroVeGA Survey 2

**Figure 13:**
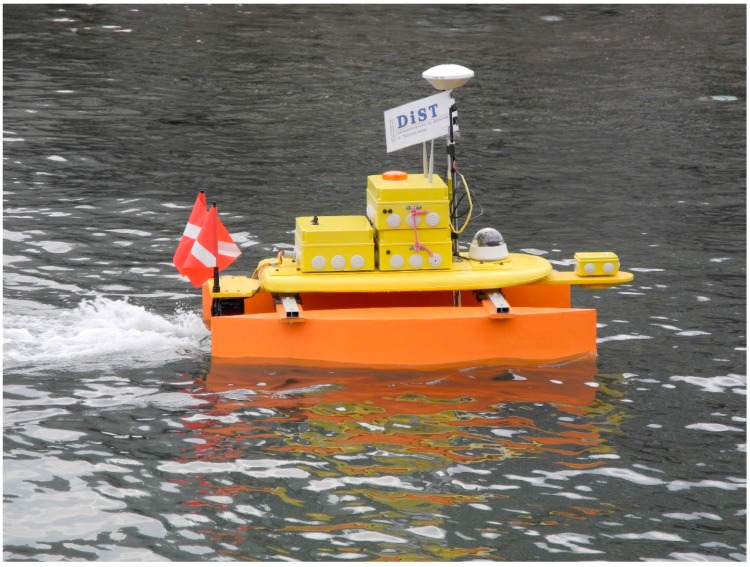
MicroVeGA in action.

The second bathymetric survey of MicroVeGA drone was carried out along the Posillipo Hill (Naples, Italy) coast in the nearshore area (0–10 m depths) of Marechiaro harbour, using Prototype #2. The instruments on board the second prototype were completely contained in a waterproof case and the obstacle-detection system was installed on a waterproofed wooden support on the drone bow, in addition, the catamaran’s hulls were made of marine plywood (Figure 13), which widened the hull and lengthened the bearing surfaces side, thus increasing the stability of the drone in navigation by decreasing the pitch and roll movements (Table 4). In addition, the largest volume of the hulls, increasing the displacement, helped to improve the available payload (Table 4).

**Figure 14:**
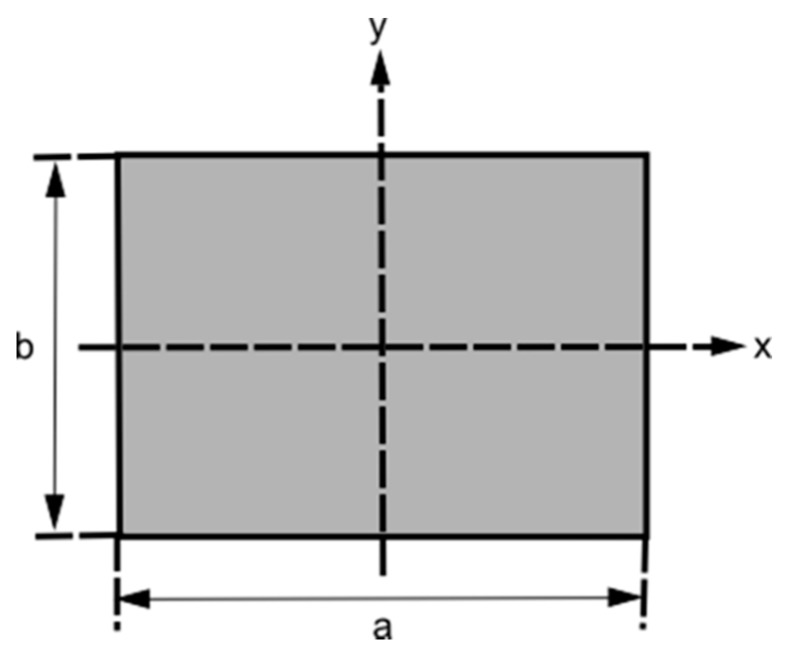
Schema of a rectangular vessel.

The transverse stability of the hull, in a catamaran like MicroVeGA, increases with the increase in the bearing surface on the water. In fact, while the longitudinal stability counteracts the pitching movements (the “fluctuations” of the vessel from bow to stern), the transverse stability counteracts the rolling motion (the lateral “oscillations” of the vessel). MicroVeGA can be approximated to a rectangular water plane, and the transversal (j_x_) and longitudinal (j_y_) moments of inertia, as shown in Figure 14, can be calculated as being equal to [18]:

(5) $$j_{x}=\frac{a\cdot b^{3}}{12}$$

(6) $$j_{y}=\frac{b\cdot a^{3}}{12}$$

Therefore in this version, the increase in the transverse and longitudinal stability increased the navigation safety (Table 4).

**Table 4:** Comparison between physical characteristics of Prototypes #1 and #2.

Prototype	Width (cm)	Length (cm)	J_x_	J_y_	Payload (kg)
MicroVeGA #1	72	92	0.029	0.047	12
MicroVeGA #2	86	120	0.064	0.124	22

**Figure 15:**
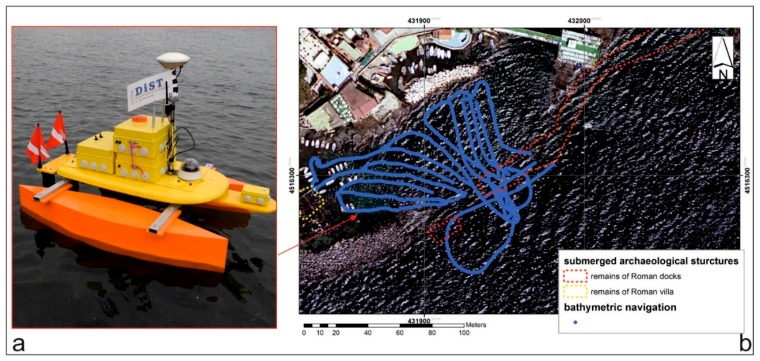
(**a**) MicroVeGA drone prototype #2 used during the survey; (**b**) navigation lines of bathymetric survey in blue and submerged archaeological structures in red.

The site of the second survey, was a port in the 1st century AD and several remains of a dock [19] are still present (red dashed line in Figure 15). MicroVeGA passed over these remains thanks to a few centimeters of draught.

The navigation of the bathymetric survey (Figure 14b) had a linear development of about 1500 m, with a distance between the navigation lines of about 5 m. In this survey, the tool that manages the inertial platform measurements eliminated 10% of depth measurement, due to the transition of some boats during the survey.

**Figure 16:**
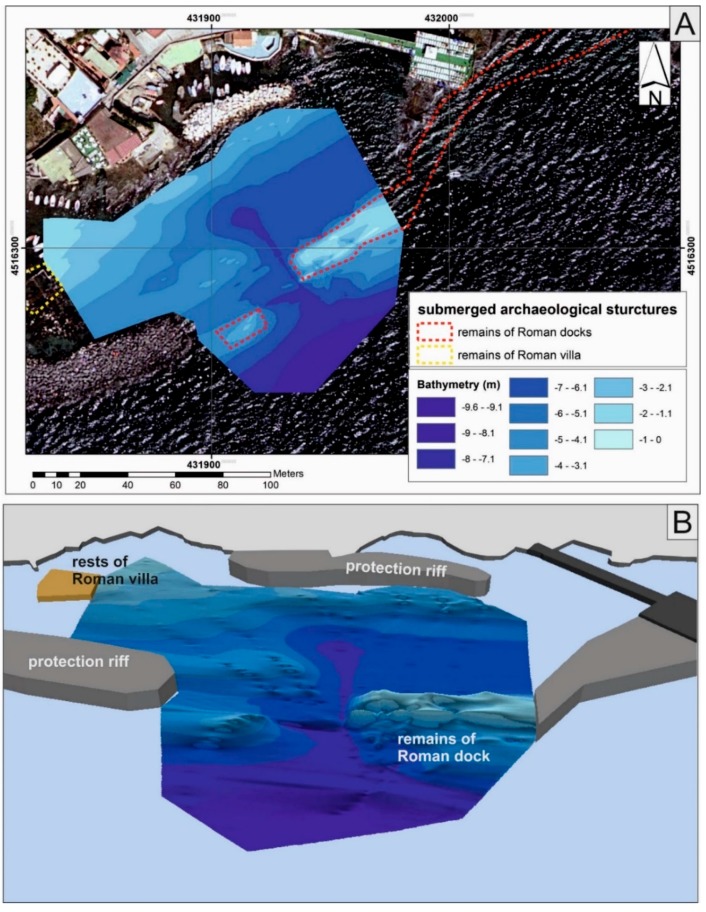
(**a**) 2D visualization of the sea floor digital model of the study area—Marechiaro harbour along Posillipo Hill (Naples, Italy); (**b**) 3D visualization of the same sea floor.

3D data were processed in ARCGIS, using the Geostatistical Analysis tool. The interpolation of the bathymetric data, through the IDW interpolator, transformed the point measurements into continuous measurements. The final product is a sea floor digital model of the area (Figure 16).

## 4. Conclusions

We have described a prototype of a marine drone optimized for very shallow water, which enables bathymetric surveys to be performed in areas that are not feasible for traditional boats. In the two study cases described in this paper, the various underwater structures would have created many navigation difficulties, if MicroVeGA had not had only a few centimeters of draught.

The experiments performed in the two coastal sites showed that integrating several existing technologies improved the final performance and the quality of the acquired data. The development of a specific software application (Trackstar) improved the accuracy of all the measured data, thus increasing the instruments’ performance.

Trackstar improves survey accuracy using the inertial platform which extended the survey duration but always guaranteed a high quality control of measurements. In fact, during the planning phase of the survey, we established the survey precision parameter ssp as a function of survey scale, depth and survey target, reducing the attitude errors, as demonstrated in the Marechiaro survey where the effect of the sailing boats was deleted. The control of the speed and the possibility of navigating at a reduced speed also ensured a greater measurement accuracy.

In addition, the safety performance of the operation was improved by integrating the temperature sensor with the ultrasonic sensor, thus increasing the accuracy in the measurements of the distance from the obstacles, as demonstrated in Table 4.

Another important characteristic of this project is the low technology risk philosophy, guaranteed by the spiral model used to manage the drone construction phases. In fact, we had carried out a bathymetric survey in the Sorrento Marina Grande site, already using the first prototype.

Prototypes #1 and #2 provide the basic requirements of practicality and economy. Practicality is clear from the ease of performing the measurements (small footprint, highly portable, ultra lightweight and easy manoeuvrability). Low costs were achieved by assembling and integrating existing systems.

Finally, MicroVeGA is equipped not only with bathymetric sensor but also with an underwater camera which provides an overview of the investigated seabed and the surrounding underwater environment.

**Figure 17:**
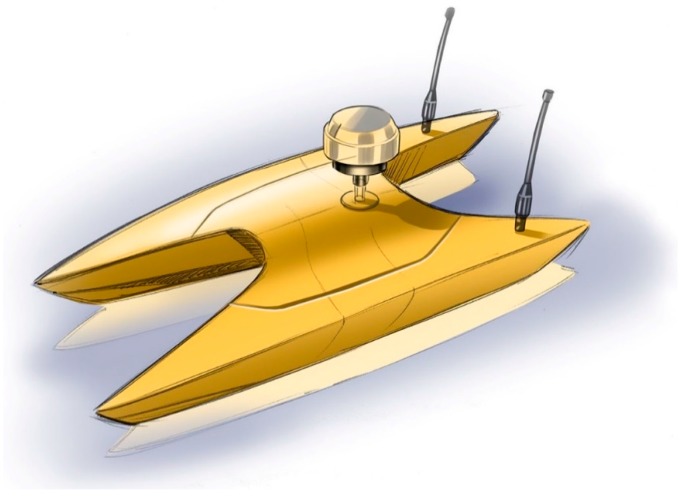
An industrial mock up in fiberglass.

In the next (*i.e.*, third) phase of this project, the experience obtained in the current development phases will be used to design morpho—bathymetric surveys in critical areas. Future plans include new survey strategies and an industrial mock up in fiberglass (Figure 17).
